# Utilization of EHRs for clinical trials: a systematic review

**DOI:** 10.1186/s12874-024-02177-7

**Published:** 2024-03-18

**Authors:** Leila R. Kalankesh, Elham Monaghesh

**Affiliations:** 1 Tabriz Health Services Management Research Center, Tabriz University of Medical Sciences, Tabriz, Iran; 2 Student Research Committee, Tabriz University of Medical Sciences, Tabriz, Iran; 3 Department of Health Information Technology, School of Management and Medical Informatics, Tabriz University of Medical Sciences, Tabriz, Iran

**Keywords:** Electronic Health Record, EHR, Clinical trials

## Abstract

**Background and objective:**

Clinical trials are of high importance for medical progress. This study conducted a systematic review to identify the applications of EHRs in supporting and enhancing clinical trials.

**Materials and methods:**

A systematic search of PubMed was conducted on 12/3/2023 to identify relevant studies on the use of EHRs in clinical trials. Studies were included if they (1) were full-text journal articles, (2) were written in English, (3) examined applications of EHR data to support clinical trial processes (e.g. recruitment, screening, data collection). A standardized form was used by two reviewers to extract data on: study design, EHR-enabled process(es), related outcomes, and limitations.

**Results:**

Following full-text review, 19 studies met the predefined eligibility criteria and were included. Overall, included studies consistently demonstrated that EHR data integration improves clinical trial feasibility and efficiency in recruitment, screening, data collection, and trial design.

**Conclusions:**

According to the results of the present study, the use of Electronic Health Records in conducting clinical trials is very helpful. Therefore, it is better for researchers to use EHR in their studies for easy access to more accurate and comprehensive data. EHRs collects all individual data, including demographic, clinical, diagnostic, and therapeutic data. Moreover, all data is available seamlessly in EHR. In future studies, it is better to consider the cost-effectiveness of using EHR in clinical trials.

## Introduction

Clinical trials are of high importance for medical progress [1]. Well designed and well-executed clinical trial studies provide the foundational data for evidence-based medicine [2], which are the standard for evaluating the benefits and harms of medical interventions [3]. Numerous factors lead to the success of clinical trials, such as appropriate trial design(e.g. randomization, blinding, and controls), thorough training of research staff, and recruitment of an adequate sample size by identifying and enrolling qualified participants in a timely manner [4, 5] and maintaining good participation through study completion [2, 6].

Strategic selection of study sites with access to suitable patient populations can optimize recruitment. Moreover, developing practical yet scientifically sound protocols through careful planning and analysis helps ensure trials are completed in an accurate and cost-effective manner [2]. Traditionally, many trials have relied heavily on physician referrals to identify and attract potential participants [7]. While essential, sole dependence on this approach has limitations including referral bias and logistical challenges that could hamper recruitment. To strengthen the recruiting process, manually reviewing patient’s electronic records to identify and diagnose eligible candidates for clinical trials has become a standard practice [8]. However, this manual chart review method is often time-consuming and resource-intensive [9].

To modernize clinical recruitment and conduct, new tools have been developed that enable data-driven insights into patient populations within EHR systems [10]. In fact, to digitalize processes, the TransCelerate e-Resource initiative, launched in January 2016, aims to facilitate understanding the e-resource landscape and the optimal use of electronic data resources to improve clinical science and clinical trial implementation for stakeholders. The eSource initiative also aligns well with other TransCelerate initiatives designed to help modernize trial execution and ways to enroll patients in clinical trials [38].

EHR systems contain comprehensive demographic, medical and treatment history collected during routine care, which offer potential to efficiently pre-scan, identify and recruit appropriate patients for clinical trials [11–14]. Specifically, recruiting patients through the EHR allows pre-assessment of eligibility criteria, selection of targeted population, and automated outreach to participants [15]. EHRs also provide ongoing access to detailed patient data that may decrease redundant measurements and data collection during trials [12]. Overall, EHR-enabled recruitment and workflow processes have potential to make clinical trials more cost-effective and feasible [11]. This study conducted a systematic review to identify the applications of EHR in supporting and enhancing clinical trials.

## Method

### Study design

This systematic review followed the Preferred Reporting Items for Systematic Reviews and Meta-Analyses (PRISMA) guidelines.

### Literature search

A systematic search of PubMed was conducted on 12/3/2023 to identify relevant studies on the use of EHRs in clinical trials. The search included a combination of Medical Subject Headings (MeSH terms) and keywords related to electronic health records (EHR OR electronic medical record) AND clinical trials. The search was limited to title and abstract fields. No date or language limits were applied. The specific Boolean search syntax was:

- ("EHR"[Title/Abstract] OR "Electronic health record"[Title/Abstract] OR "Electronic health records"[Title/Abstract] OR "EMR"[Title/Abstract] OR "Electronic medical record"[Title/Abstract] OR "Electronic medical records"[Title/Abstract]) AND (clinical trial* [Title/Abstract]).

Reference lists of included studies were hand-searched to identify additional relevant articles. The search was performed without any time limit.

### Eligibility criteria

Studies were included if they (1) were full-text journal articles, (2) were written in English, (3) examined applications of EHR data to support clinical trial processes (e.g. recruitment, screening, data collection). Reviews, letters, abstracts, editorials and other non-research studies were excluded.

### Study selection and data extraction

Two researchers (EM and LRK) independently screened titles and abstracts of retrieved records to identify potentially eligible studies. After obtaining full texts of potential articles, the two investigators independently assessed eligibility based on predefined criteria. Disagreements were resolved through discussion and consensus. A form was used by two reviewers to extract data on: study design, EHR-enabled process(es), related outcomes, and limitations.

### Evidence synthesis

A qualitative synthesis was conducted summarizing key outcomes and limitations of included studies grouped by the EHR-enabled process examined. The study authors met regularly to discuss consensus on findings.

## Result

**Fig. 1:**
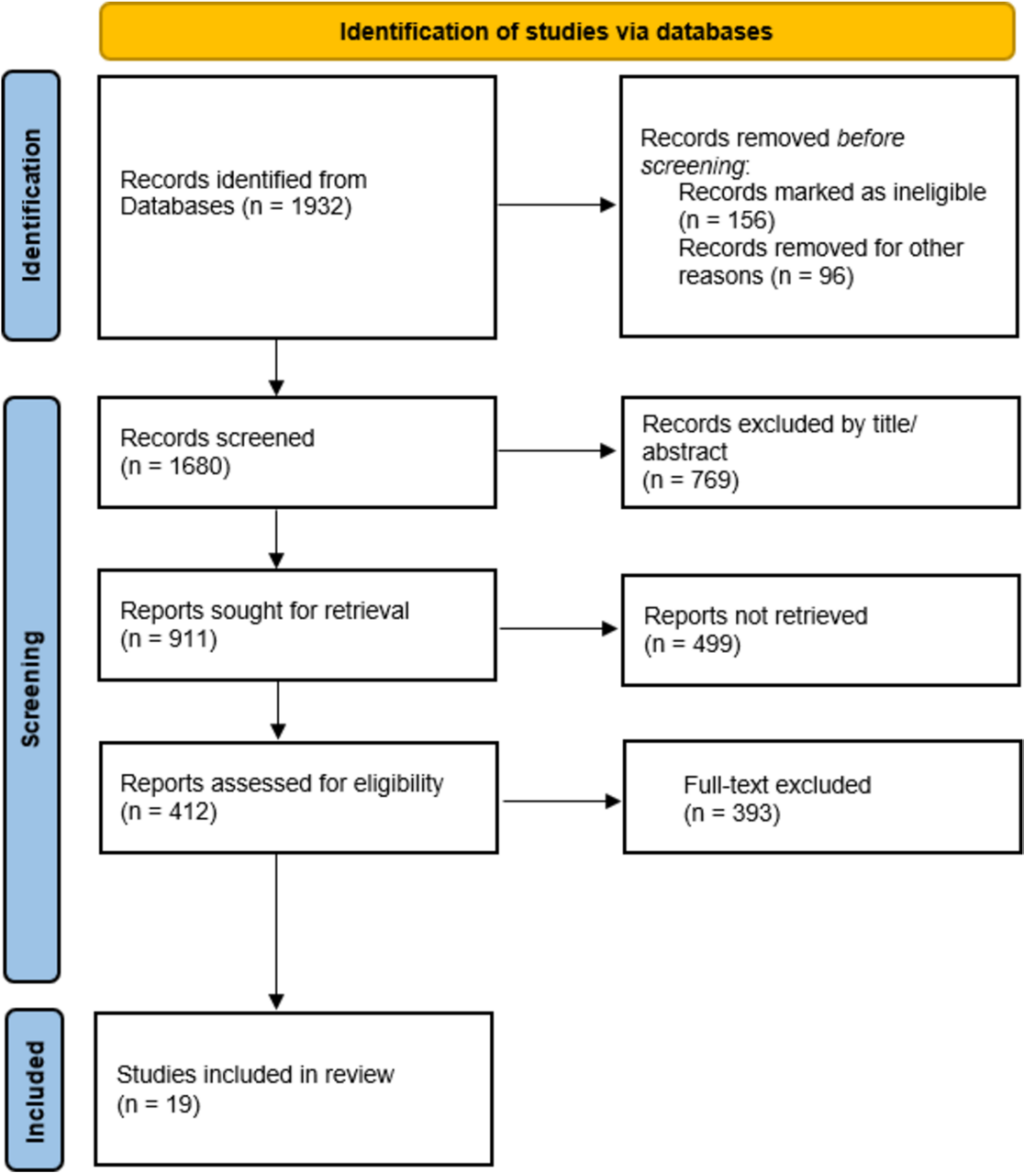
PRISMA flow diagram illustrating study selection for utilization of EHRs in Clinical Trials

The systematic literature search yielded 2161 records, out of which 312 were selected for full-text review after screening titles and abstracts. After conducting a thorough review of the full-texts and resolving disagreements regarding 2 articles, a total of 19 studies that met the predefined eligibility criteria were included in the final qualitative synthesis (Fig. 1).

### Characteristics of included studies

The key characteristics of the 19 included studies are summarized in Table 1. The studies were published in a variety of international journals, with the majority (14/19) from the United States. The remaining studies originated from China, Switzerland, Germany, Belgium, and Finland. The sample sizes ranged from 165 to 5,529,407 patients.

**Table 1:** Summary of the basic characteristics of the studies included in the systematic review

Author, year	Country	Aim of study
Mohammad B Ateya 2016 [16]	USA	To quantify the proportion of eligibility criteria that can be addressed with data in electronic health records and to compare the content of eligibility criteria in primary care with previous
Ariel Beresniak 2016 [17]	Switzerland	To evaluate the efficacy of EHR4CR (Electronic Health Records for Clinical Research) solutions in comparison to current practices, from the viewpoint of clinical trial sponsors.
Philipp Bruland 2016 [18]	Germany	To determine the most commonly used data elements in clinical trials and their availability in hospital EHR systems.
Jake Carrion 2018 [19]	USA	To examine the observed benefits and drawbacks of using Electronic Health Record (EHRs) to enhance various patient-centered aspects of the clinical trial process, specifically its potential to improve patient recruitment, patient retention, and data collection
GeorgesDe Moor 2015 [20]	Belgium	To describe the EHR4CR project which aims to demonstrate a scalable, widely acceptable and efficient approach to interoperability between Electronic Health Record (EHR) systems and clinical research systems.
Peter J. Embi 2005 [21]	Ohio	To determine if the use of EHR-based Clinical Trial Alert (CTAs) could enhance physicians’ participation in subject recruitment and increase physician-generated recruitment rates for an ongoing clinical trial.
Natalie C. Ernecof 2018 [22]	USA	To develop an EHR phenotype for identifying patients with late-stage dementia for a clinical trial of palliative care consultation.
Jae Hyun Kim 2021 [23]	USA	To evaluate the impact of eligibility criteria on recruitment and observable clinical outcomes of COVID-19 clinical trials using EHR data
Jeffrey Kirshner 2021 [24]	USA	To facilitate identification of clinical trial participation candidates, the researchers developed a machine learning tool that automates the determination of a patient's metastatic status, on the basis of unstructured EHR data.
Niina Laaksonen 2021 [25]	Finland	To evaluate the accuracy of a commercially available EHR Research Platform, “InSite”, in identifying potential trial participants from the EHR system of a large tertiary care hospital.
Mengyang Li 2021 [26]	China	To develop a patient-screening tool for clinical research using openEHR to address concept mismatch and enhance query performance.
Stéphane M. Meystre 2019 [27]	USA	To assess the feasibility of determining a patient's eligibility for a sample of breast cancer clinical trials by automatically mapping coded clinical trial eligibility criteria to the corresponding clinical information extracted from text in the EHR.
Riccardo Miotto 2015 [28]	USA	To develop a cost-effective, case-based reasoning framework for clinical research eligibility screening by reusing only the EHRs of a minimal number of enrolled participants to represent the target patient for each trial under consideration.
Sarah J Nelson 2021 [29]	USA	To develop a service line for extracting study population estimates from EHR systems to aid in selecting enrollment sites for multicenter clinical trials.
Yizhao Ni 2019 [30]	USA	To evaluate the impact of Automated Clinical Trial Eligibility Screener (ACTES) on the institutional workflow, both prospectively and comprehensively.
Emily C. O’Brien 2021 [31]	USA	To describe the current site-level processes for utilizing the EHR to identify and screen potential participants for an ongoing clinical trial.
James R. Rogers 2021 [32]	USA	To identify the extent of main clinical differences between clinical trial participants and nonparticipants using a combination of electronic health record and trial enrollment data.
Yingcheng Sun 2021 [33]	USA	To systematically estimate the representativeness of the population in clinical trials using EHR data during the early design stage.
Lindsay P. Zimmerman 2018 [34]	USA	To present a novel strategy for recruiting underrepresented, community-based participants for pragmatic research studies that leverage routinely collected EHR data.

### Clinical Trial Processes and Outcomes

Nineteen studies examined the impacts of EHR use on clinical trial processes and outcomes. Table 2 summarizes the key findings on EHR applications for recruitment, screening, data collection, and trial design. Overall, the included studies consistently demonstrated that utilization of EHR data improved clinical trial feasibility and efficiency in the following ways:

**Table 2:** Application cases of the EHR in Clinical Trials processes and outcomes reported in the included studies

Author, year	Application cases	Key findings
Mohammad B Ateya 2016 [16]	EHRs as a potential source for assessing patients' eligibility for enrollment in clinical trials.	Careful design of EHR systems that include data elements representing the content categories will facilitate integration with clinical trial management systems, and improve patient care and clinical research.
Ariel Beresniak 2016 [17]	Reuse of EHR data for executing clinical trials.	Improving clinical trial design and execution using the EHR4CR platform would provide significant benefits for pharmaceutical industry, which serves as the primary sponsor of clinical trials in Europe, and other regions.
Philipp Bruland 2016 [18]	Utilizing EHR data for secondary purposes beyond direct patient care.	Common data elements relevant to clinical trials were identified and their availability within hospital information systems were assessed.
Jake Carrion 2018 [19]	Improvement of patient recruitment, patient retention, and data collection through using the EHR.	Leveraging EHR tools can enhance the way clinical trials are planned and executed.
GeorgesDe Moor 2015 [20]	Streamlining the optimization of clinical trial protocols and facilitating patient identification and recruitment.	EHR4CR could be well placed to deliver a sound, useful and well accepted pan-European solution for the reuse of hospital EHR data to support clinical research studies.
Peter J. Embi 2005 [21]	Solution to the common problem of inadequate trial recruitment.	Use of an EHR-based CTA led to significant increases in physicians’ participation in and recruitment rates to an ongoing clinical trial.
Natalie C. Ernecof 2018 [22]	A novel method using an EHR phenotype plus brief medical record review is effective to identify hospitalized patients with late-stage dementia.	In health care systems with similar clinical data warehouses, this method may be applied to serious illness populations to improve enrollment in clinical trials of palliative care or to facilitate access to palliative care services.
Jae Hyun Kim 2021 [23]	Recruitment and observable clinical outcomes of COVID-19 clinical trials.	This research demonstrated the potential of using the EHR data of COVID-19 patients to inform the selection of eligibility criteria and their thresholds, supporting data-driven optimization of participant selection towards improved statistical power of COVID-19 trials. This method promises to improve feasibility and efficiency for COVID-19 clinical trial recruitment.
Jeffrey Kirshner 2021 [24]	This tool infers from unstructured EHR data with high accuracy and high confidence in more than 75% of cases, without requiring additional manual review.	This tool could mitigate a key barrier for patient ascertainment and clinical trial participation in community clinics.
Niina Laaksonen 2021 [25]	Identify potential trial participants from the EHR system of a large tertiary care hospital.	The EHR query resulted in a larger patient count than the manual query. Searching for patients with the EHR Research Platform can help identify potential trial participants from a hospital's EHR system by limiting the number of records to be manually reviewed.
Mengyang Li 2021 [26]	Utilizing EHR for designing a patient screening tool for clinical research that provides high-level expressions and improves query performance.	It not only helps solve concept mismatch but also improves query performance to reduce the burden on researchers.
Stéphane M. Meystre 2019 [27]	Deploying NLP to extract clinical trial eligibility criteria from EHR clinical notes.	By leveraging machine learning-based NLP, this system can automatically discover eligible patients for clinical trials with good accuracy and reduce the workload of human screening for clinical trials.
Riccardo Miotto 2015 [28]	Identifying eligible patients for clinical trials by processing data from EHR.	Potential candidates for clinical trials can be identified efficiently through applying case-based reasoning model on EHR data.
Sarah J Nelson 2021 [29]	Producing reliable counts of potentially eligible study participants.	The RIC EHR cohort evaluation process is efficient and useful for potential sites for multicenter trials.
Yizhao Ni 2019 [30]	Enhancing the efficient identification of patients through pre-screening based on EHRs, streamlining patient recruitment workflow and increasing enrollment in clinical trials.	By leveraging NLP and machine learning technologies, the Automated Clinical Trial Eligibility Screener (ACTES) demonstrated good capacity for improving efficiency of patient identification. The quantitative assessments demonstrated the potential of ACTES in streamlining recruitment workflow and improving patient enrollment. The post evaluation surveys suggested that the system was a good computerized solution with satisfactory usability.
Emily C. O’Brien 2021 [31]	EHR screening was commonly used for recruitment in a cardiovascular outcomes trial.	The EHR is effective in finding potential trial participants.
James R. Rogers 2021 [32]	Using a combination of EHR and trial enrollment data.	Combining trial enrollment data with EHR data may be useful for better understanding of the generalizability of trial results.
Yingcheng Sun 2021 [33]	Using the EHR data to assess the clinical trials population representativeness, providing data-driven metrics to inform the selection and optimization of eligibility criteria.	The EHRs data are useful for estimating the population representativeness of clinical trial study.
Lindsay P. Zimmerman 2018 [34]	Direct outreach to community participants, while utilizing EHR data for clinical information and follow-up, allows for efficient recruitment and follow-up strategies. Feasibility of eligibility verification and automated follow-up.	Improvement of recruitment efficiency and engagement traditionally underrepresented individuals in research.

- Recruitment: 19 studies evaluating EHR-enabled recruitment have reported increased enrollment efficiency compared to standard practices.
- Screening: In 5 studies, EHR pre-screening excluded patients prior to full eligibility screening, reducing unnecessary procedures.
- Data collection: In 3 studies using EHR data reduced data collection costs compared to standard methods.
- Trial Design: In one study examining this application, EHR data informed optimization of eligibility criteria to improve statistical power for a COVID-19 trial.

### Purposes of using EHR

The most frequent application of EHR data was to identify and recruit eligible participants into clinical trials. By containing diverse information on demographics, clinical history, diagnoses, and more, EHRs allowed pre-screening and outreach to potential candidates that met enrollment criteria. In several studies, EHR data was leveraged for secondary research purposes including data collection, data analysis and optimizing trial design [16, 27–30, 38]. Specifically, one study utilized EHR data from COVID-19 patients to inform eligibility criteria selection and improve statistical power for COVID-19 trials [23]. Overall, the primary use case was to enable secondary research applications of EHR data beyond routine clinical care to facilitate clinical trial processes. Key limitations of these applications included potential for selection bias, generalizability concerns in single health system populations, and heterogeneity in methods and endpoints assessed across studies. Further investigation using standardized methodology is needed to realize the full potential of EHR-enabled clinical research.

## Discussion

This systematic review aimed to identify applications and impacts of electronic health record (EHR) use in clinical trials. The included studies demonstrated EHR data has been leveraged to serve various key functions, including identifying eligible participants, facilitating recruitment, enabling data collection and analysis, and optimizing trial design.

In one study, EHR data was from 59639 patients who encountered health care system. The results showed that the EHR data could be used as a promising clinical tool to assist physicians in early identification of patients suitable for palliative care counseling [35]. Although this study used EHR for therapeutic purposes, it can be concluded that EHR data is very effective in identifying individuals with any target.

Another study found that primary care electronic health record data could be used effectively to identify patients who have been prescribed specific medications and patients who are potentially experiencing drug side effects [36]. In general, based on the results of this study, EHR can also be utilized in clinical trials for purposes other than patient care and in particular for the secondary use of this tool. In fact, according to the studies [20, 28–32], the use of EHR serves various purposes in clinical trials, including identifying eligible participants, facilitating their recruitment and analyzing patient data to assess outcomes and measure the safety and efficacy of the intervention.

EHRs can be used as a database for the use of data needed in clinical trials. For example, a study in Brazil used EHR data to obtain benchmark for stroke patients [37].

According to the results of the present study, the use of EHR in conducting clinical trials is very helpful. Therefore, it is better for researchers to use EHR in their studies for easy access to more accurate and comprehensive data. EHRs collects all individual data, including demographic, clinical, diagnostic, and therapeutic data. So that all data is available seamlessly. Real-time access to patient data directly from EHRs could eliminate the need for manual data entry, minimizing errors and ensuring data integrity.

Moreover, EHRs enable the seamless integration of clinical trial data with other relevant health information, providing a more comprehensive picture of patient health and facilitating the evaluation of long term outcomes. In future studies, it is better to consider the cost-effectiveness of using EHR in clinical trials. Because due to the increasing use and effectiveness of using EHR in clinical trials, its cost-effectiveness should also be determined. Also, conducting such research would be useful for the wider scientific community. Also, in future studies, many metrics can be investigated and reported to reflect the effectiveness of EHR for patient registration. Also, some statistics can be shown to illustrate this.

One of the limitations of the present study was the lack of access to some databases due to sanctions. Another limitation is the lack of a similar study that comprehensively examines the role and effectiveness of EHR in clinical trials. There are also a small number of studies that have examined the effectiveness, how the EHR is used, and its uses in clinical trials.

Another limitation is related to the comparison of the studies included in this study, considering that the EHR system used in different countries, even in each country, is very different in many aspects, including the type of system used, the culture of each country, the level of EHR implementation, technical infrastructure, etc. Therefore, the comparison between systems was one of the limitations of this study.

## Conclusions

According to the results of the present study, it can be concluded that EHR in clinical trials is used for various purposes. While promising, several limitations should be considered when interpreting the evidence. Many EHRs may rely on single health system populations, limiting generalizability of findings. Heterogeneity in methods and endpoints used to evaluate the same EHR processes is another issue to be considered. Additional limitations included potential for selection and referral bias. More research is needed to develop standardized methodology and reporting for EHR-enabled clinical trials. Future directions of the research should be to optimize EHRs for supporting clinical trials. This may be realized through enhanced interoperability and data sharing between EHR systems to facilitate multi-site and diverse patient populations trials and expand access to diverse patient populations beyond single health systems. Standardization of data formats, development of shared platforms, and policies enabling access are needed. Integration of clinical trial-specific modules into EHRs is required to simplify participant screening, recruitment, enrollment, and data collection. This could include dashboards, automated alerts, and documentation templates. Advanced analytics and machine learning applied to EHR data can also be a part of agenda for future research. Stronger privacy protections and cybersecurity measures should be in place to securely operationalize EHR data for research while maintaining patient confidentiality.

There is also gap in cost-effectiveness studies to quantify financial benefits and guide investments in EHR-enabled research infrastructure.

## Data Availability

Not applicable.
